# Sex-Specific Size Analysis of Carpal Bones: Implications for Orthopedic Biomedical Device Design and Therapy Planning

**DOI:** 10.3390/life14010140

**Published:** 2024-01-18

**Authors:** Malte Asseln, Valentin Quack, Roman Michalik, Björn Rath, Frank Hildebrand, Filippo Migliorini, Jörg Eschweiler

**Affiliations:** 1 Department of Biomechanical Engineering, University of Twente, 7522 NB Enschede, The Netherlands; 2 Department of Orthopaedics, Trauma, and Reconstructive Surgery, RWTH Aachen University Hospital, 52074 Aachen, Germany; 3 Department of Orthopaedic Surgery, Klinikum Wels-Grieskirchen, 4600 Wels, Austria; 4 Department of Orthopaedic and Trauma Surgery, Academic Hospital of Bolzano, 39100 Bolzano, Italy; 5 Department of Trauma and Reconstructive Surgery, BG Hospital Bergmannstrost, 06112 Halle (Saale), Germany; joerg.eschweiler@bergmannstrost.de

**Keywords:** wrist, morphology, biomedical device design, therapy planning, biomechanics

## Abstract

Consideration of the individual carpal bone characteristics of the wrist plays a key role in well-functioning biomedical devices and successful surgical procedures. Although geometric differences and individual bone sizes have been analyzed in the literature, detailed morphologic descriptions and correlations covering the entire wrist reported in a clinical context are lacking. This study aimed to perform a comprehensive and automatic analysis of the wrist morphology using the freely available “Open Source Carpal Database” (OSCD). We quantified the size of each of the individual carpal bones and their combination. These sizes were extracted in n = 117 datasets of the wrist of the OSCD in anatomical directions and analyzed using descriptive statics and correlation analysis to investigate the morphological characteristics under sex-specific aspects and to provide regression plots and equations to predict individual carpal bone sizes from the proximal and distal row dimensions. The correlations in the proximal row were higher compared to the distal row. We established comprehensive size correlations and size rations and found that there exist statistical differences between sex, particularly of the scaphoid. The regression plots and equations we provided will assist surgeons in a more accurate preoperative morphological evaluation for therapy planning and may be used for future anatomically inspired orthopedic biomedical device designs.

## 1. Introduction

The human wrist joint contains eight complexly shaped carpal bones, positioned between the two forearm bones the radius and ulna and the five metacarpal bones. In the context of well-functioning orthopedic biomedical devices, such as fusion plates, compression implants, osteotomy plates, prostheses, and external fixators, and successful therapy planning, such as for physiological/anatomical reconstruction, it is important to first understand and quantify the morphological characteristics and then, second, to relate the geometry to biomechanics such as joint forces and motion. This knowledge is a prerequisite for truly anatomically inspired designs and target definitions in surgical planning. It also helps to understand to what extent patient-specific, subpopulation-based (e.g., differentiation by sex), or population-based approaches are needed.

Morphology is the study of the form and structure of organisms and their specific structural features [1]. The morphology of the wrist joint has been previously studied in the literature, and differences in motion behavior and shape have been reported, for example, between sex [2,3,4,5,6,7,8,9] and ethnicity [10]. Crisco et al. reported that carpal motion seems to be similar in females and males but there are differences in the location of the rotation axes [3]. The rotational axis defines the position of the overall resulting axis of rotational movement of the wrist joint in two planes (radial–ulnar deviation (RUD) and flexion–extension movement (FEM)). In particular, the rotation axes of the carpal bones in females are located more proximally compared to the location in males [11]. Differences in the rotation axis location may be due to differences in the bone size of the different carpal bones, as opposed to some functional differences [3]. This implies a sex specificity for females and males. Besides sex specificity, individual anatomical variations might cause different motion behaviors or bony interactions (kinematic interaction between the different carpal bones based on individual bony contact), too. Crisco et al. studied the wrist morphology under sex-specific aspects and reported that carpal bone sizes increase isometrically with increasing volume [3]. However, they used the dimensions in the directions of the principal axes as the outcome of principal component analysis, which makes it difficult to interpret their results clinically. Although the morphology of the carpal bones has already been studied in the literature, there is a lack of comprehensive analysis and presentation of the results on size variations, size correlations, and size ratios in anatomical terms.

This study aimed to comprehensively analyze the different carpal bone dimensions under sex-specific aspects for a larger number of cases and to provide anatomical correlations to predict individual carpal bone sizes from the proximal and distal row dimensions as a guide for anatomically driven orthopedic biomedical device designs and surgical planning. This paper addresses the problem of automatic bone geometry extraction and statistical analysis considering sex differences in carpal bone size. The results obtained may also help to extend the currently available anatomical knowledge to ultimately describe the relationship between bone shapes and wrist function.

## 2. Materials and Methods

### 2.1. Subjects and Data Acquisition

We used the “Open Source Carpal Database” (OSCD) for our investigation [12]. The OSCD includes anatomical information on the individual carpal bone geometries from 90 healthy subjects (120 wrists) and the carpal bone kinematics in 1215 unique wrist positions. The datasets are freely available online in the OSCD (https://simtk.org/projects/carpal-database, accessed on 6 June 2020) [12]. The datasets available in the OSCD were acquired from different previous studies [3,11,13,14,15,16,17,18,19,20,21,22]. The geometrical information is available as bone surface models that have been segmented and reconstructed from computed tomography (CT) scans (Lightspeed 16; GE Medical, Milwaukee, WI) [12,17,23]. The CT scan resolutions differed between the datasets, and ranged from 0.2 × 0.2 mm to 0.4 × 0.4 mm in the transverse plane of the hand and 0.625 to 1 mm along the axis of the forearm [12]. Digital models of the outer cortical surface of the radius, ulna, the eight carpal bones, and the five metacarpals are available in the database and were obtained from neutral posture CT images using Mimics v12–19 (Materialize, Leuven, Belgium) [12]. The neutral posture was defined as the posture where the third metacarpal bone was aligned in line with the orientation of the two forearm bones. Information on the cartilage was not available from the CT images [12]. In our study, datasets on 117 wrist joints (62 female and 55 male datasets) were included, containing eight carpal bones, the radius, and the ulna. Three datasets were incomplete and excluded: in datasets 62,641 (left) and 62,641 (right), the ulna is missing, and in dataset 97,808 (right), the trapezoid is missing.

### 2.2. Data Processing and Carpal Bone Size Analysis

**Figure 1:**
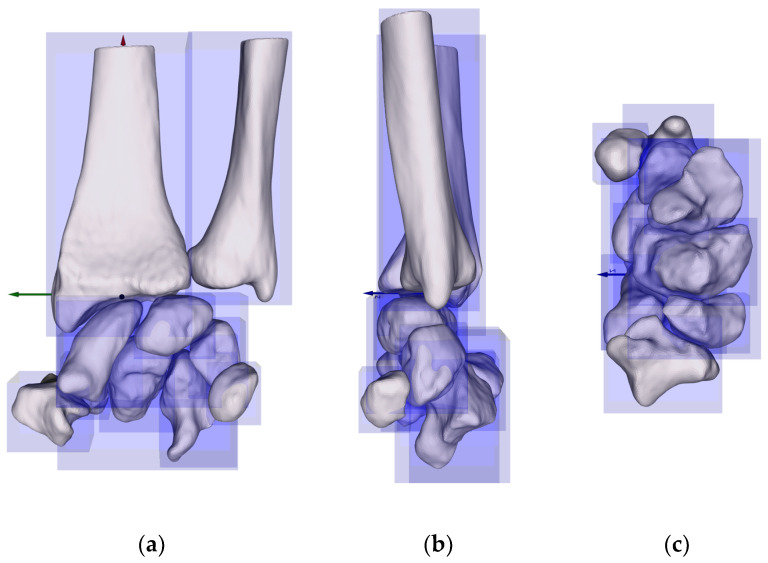
Virtual bounding boxes around each bone and the general ACS (positive x-direction (red) = distal–proximal; y-direction (green) = ulnar–radial; z-direction (blue) = dorsal–palmar) in different views: (**a**) frontal plane; (**b**) sagittal plane; (**c**) transverse plane.

Initially, the bone surface models were available in the IV (OpenInventor/ASCII) file format. These were converted for later analysis into standard polygon mesh geometries using a custom software routine written in Python in the Spyder IDE based on the open-source libraries NumPy and NumPy-stl, which used the point coordinates and indices to create lists of vertices and faces. Subsequently, the surface models were transformed from the respective CT scanner coordinate system into an anatomical coordinate system (ACS) using a rigid body transformation with the following conventions: The x-axis was defined by the long axis of the radius shaft (the positive direction was defined as running from distal to proximal). The y-axis was defined as a line perpendicular to the x-axis, originating from the center of the radial articular joint surface (the positive direction of the axis was defined as running from ulnar to radial). The z-axis was the cross-product of the other axes (dorsal to palmar orientation). The origin was the projection of the intersection of the *x*-axis direction and y-axis direction onto the distal radius surface [12]. A bounding box aligned with the ACS was calculated for each bone to measure the dimensions (Figure 1). The bounding box describes the spatial location of an object. Finally, the geometrical information (length, width, and height of the bone) was calculated in Python based on the open-source packages NumPy, trimesh, and vtkplotter for each of the eight carpal bones and the two forearm bones. The x, y, and z dimensions of the bounding box correspond to the anatomical directions distal–proximal, ulnar–radial, and dorsal–palmar, respectively.

### 2.3. Statistical Analysis

The statistical analysis included the calculation of measures of central tendency (mean) and measures of variability (standard deviation (SD)) and the plotting of boxplots and regression plots for graphical interpretation. Additionally, correlation coefficients, regression equations, and the ratios of the carpal bones were calculated. The boxplots and regression plots were created in Python using the open-source library Seaborn. Sex-specific differences were analyzed particularly using comparisons of the means, boxplots, and ratios. Furthermore, the regression of different parameters (proximal wrist bones vs. proximal row and distal wrist bones vs. distal row) was calculated. Proximal wrist bones refer to the size of the individual bones, and the proximal row refers to the proximal carpal bones as a whole. The same applies for the distal carpal bones/the distal row. An R-squared (R^2^) coefficient of determination of R^2^ = 0 indicates no correlation and R^2^ = 1 is a total correlation. Regression analysis was used to investigate the relationship between the geometry of each bone and the corresponding proximal or distal row of the carpus. The dimensions in the x-direction of the radius and ulna depend on the scan range of the CT imaging and therefore have no informative value. They were excluded from the statistical analysis.

## 3. Results

### 3.1. Carpal Bone Sizes

The geometrical dimensions of the eight carpal bones in terms of means and standard deviations are presented in Table 1. It also includes the information from Crisco et al. [3] for a direct comparison. In general, the carpal bones in the males were larger than those in the females. The analysis showed that the order from large to small of each carpal geometry parameter of the proximal row was the same in males and females. The scaphoid was the largest bone in the x-direction in the proximal row, followed by the triquetrum, lunate, and pisiform. In the y-direction, the ranking was lunate, scaphoid, triquetrum, and pisiform. In the z-direction, the ranking was scaphoid, lunate, triquetrum, and pisiform. Looking at the distal row, the hamate was the largest bone in the x-direction, followed by the capitate, trapezium, and trapezoid. In the y-direction, it was the same order, and in the z-direction, the ranking was capitate, hamate, trapezium, and trapezoid.

### 3.2. Radius, Ulna, Proximal Row, and Distal Row Sizes

The sizes of the radius, ulna, proximal row, and distal row are presented in Table 2. The average size of the proximal row and the distal row was larger in males compared to females. However, the differences in the overall width were rather small compared to the height and depth. The results revealed high standard deviations of the ulna, indicating a wide variability.

### 3.3. Boxplot Analysis

**Figure 2:**
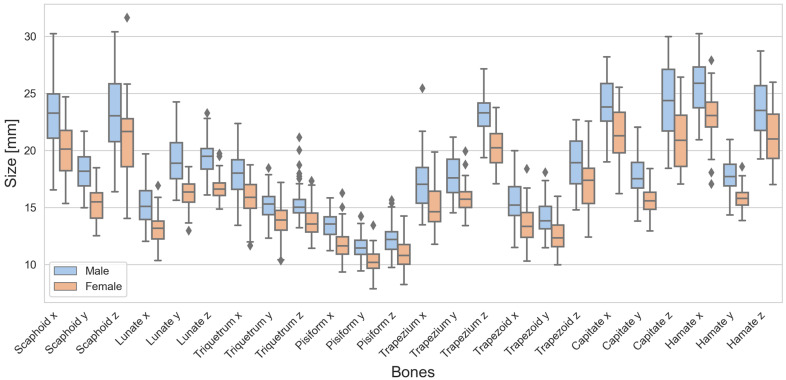
Boxplots of the carpal bones for males (blue) and females (red) separately. The black diamond markers represent outliers.

**Figure 3:**
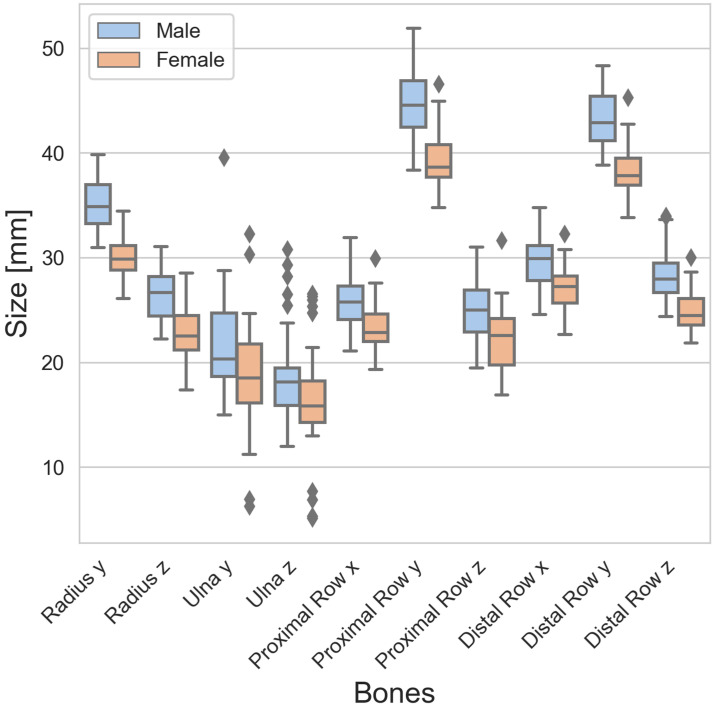
Boxplots of the radius, ulna, proximal row, and distal row for males (blue) and females (red) separately. The black diamond markers represent outliers.

There were differences in the sizes between males and females, as can be seen from the boxplots of the carpal bones (Figure 2) as well as the radius, ulna, proximal row, and distal row (Figure 3). Looking at the boxes of the carpal bones, it can be seen that they do not overlap for 7 of 24 dimensions, or in other words, the male box is completely above or below the female box, and vice versa. This indicates clear differences between males and females. Looking at the positions of the medians of the carpal bones, only for Scaphoid z and Trapezoid z do the median lines lie “inside” the box of the other sexes. This indicates that the other dimensions are likely to be different. For the radius, ulna, proximal row, and distal row, the median lines were all outside the overlap between the boxes. A wide range of dimensions expressed using long whiskers and large boxes was observed for Scaphoid x, Scaphoid z, Trapezoid z, Capitate x, Capitate z, and Hamate z. Outliers were observed specifically for Triquetrum z in the male cohort as well as Ulna z for both sexes.

### 3.4. Regression Analysis

**Figure 4:**
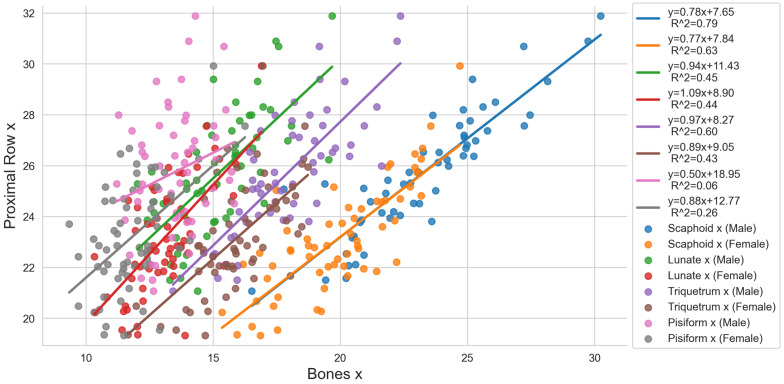

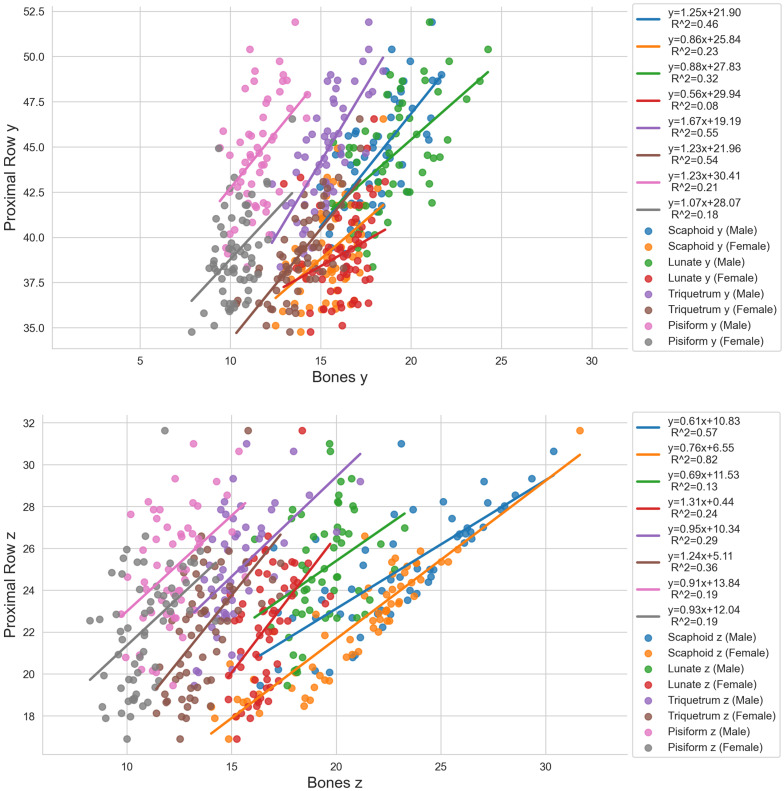
Regression analysis for the individual carpal bones of the proximal row [mm] indicated as “Bone x”, “Bone y”, and “Bone z” and the entire proximal row itself [mm] indicated as “Proximal Row x”, “Proximal Row y”, and “Proximal Row z”.

**Figure 5:**
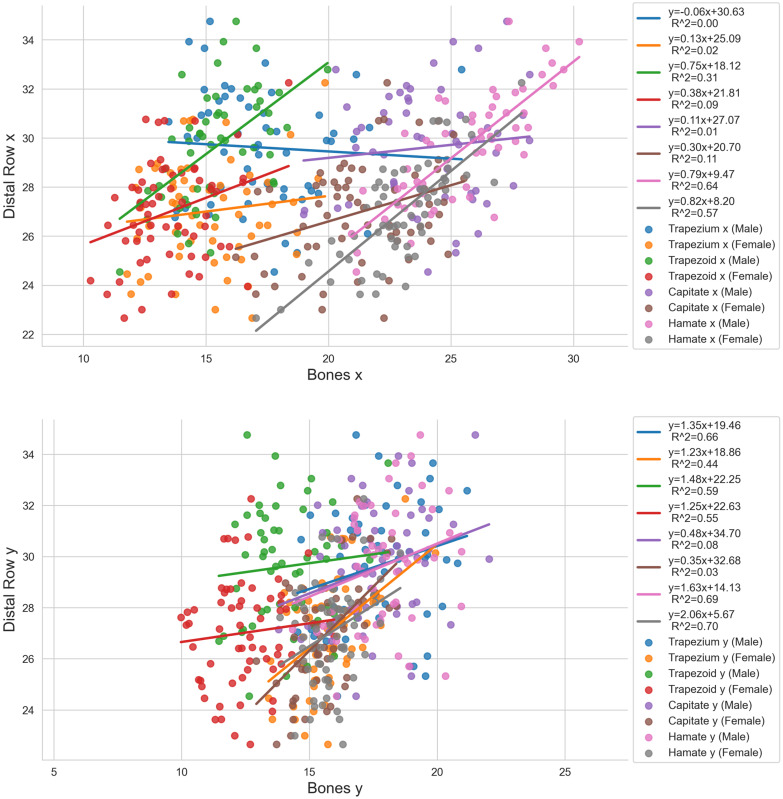

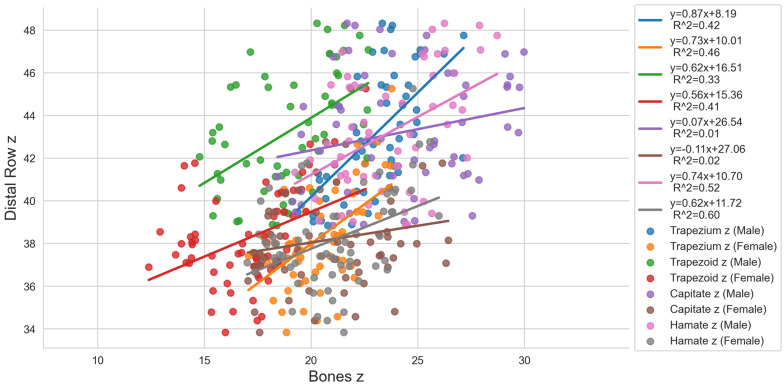
Regression analysis for the individual carpal bones of the distal row [mm] indicated as “Bone x”, “Bone y”, and “Bone z” and the entire distal row itself [mm] indicated as “Distal Row x”, “Distal Row y”, and “Distal Row z”.

The causal relationships between the individual bones of the proximal carpal row and the proximal row itself are shown in Figure 4, and those between the individual bones of the distal row and the distal row itself are presented in Figure 5. Looking at the female scaphoid of the proximal row, a very high correlation ($0.8\leq R^{2}\leq 1.0$) was observed for Scaphoid z for the proximal row in the z-direction ($R^{2}=0.82$) and a high correlation ($0.6\leq R^{2}\leq 0.79$) for Scaphoid x in the proximal row in the x-direction ($R^{2}=0.63$). Looking at the male scaphoid, we found slightly lower correlations, specifically a high correlation for Scaphoid x in the proximal row in the x-direction ($R^{2}=0.79$) and a moderate correlation ($0.4\leq R^{2}\leq 0.59$) for Scaphoid z in the proximal row in the z-direction ($R^{2}=0.57$). The results indicate a direct size relationship between the scaphoid in the x- and z-direction and the proximal row. The correlations of the lunate with the proximal row were rather low for both sexes. Partial moderate to good correlations were found for the triquetrum. Triquetrum x correlated with the proximal row in the x-direction ($R^{2}=0.60$) and Triquetrum y with the proximal row in the y-direction ($R^{2}=0.54$) for males. Looking at the females, the highest correlation was observed for Triquetrum y with the proximal row in the y-direction ($R^{2}=0.54$). The other correlations were low. The pisiform showed only very low ($0\leq R^{2}\leq 0.19$) to low correlations ($0.2\leq R^{2}\leq 0.39$) with a maximum correlation of $R^{2}=0.26$ for Pisiform x with the proximal row in the x-direction in the female datasets.

Looking at the distal row, the correlations were lower compared to the proximal row. For the trapezium, the highest correlation occurred for males in the y-direction ($R^{2}=0.66$) and were otherwise medium to not existent in the x-direction. The trapezoid showed a moderate correlation in the y-direction (male: $R^{2}=0.59$, female: $R^{2}=0.55$) and the other correlation coefficients ranged from 0.41 to 0.09. Regarding the capitate, there was no correlation existing for both sexes. Finally, the hamate correlated best for females in the y-direction ($R^{2}=0.70),$ representing a high correlation. Furthermore, high correlations occurred for Hamate x and Hamate y in the males and Hamate z in the females.

### 3.5. Carpal Bone Ratios

The carpal bone ratios are presented in Table 3. In general, the differences in the ratios between the sexes were small, as expressed by the differences in the second decimal place of the mean values. Considering the dimensions of the bounding box, this may not indicate any sex-specific shape types at a higher level. The largest ratio occurred for Hamate x/Hamate y for males (1.44±0.15) as well as females (1.46±0.11), which indicates an elongated rectangular shape in the coronal plane rather than a square. A rather square shape was observed for Scaphoid x/Scaphoid z (male: 1.04±0.24, female: 1.00±0.24) and Capitate x/Capitate z (male: 1.01±0.20, female: 1.04±0.20) for both sexes.

## 4. Discussion

Knowledge of individual morphology is an essential component of the complex wrist joint mechanism and leads to a better understanding of morphology-related functional behavior. Small bony disarrangements can lead to severe functional limitations, which means that diagnosis and therapy planning of the wrist is often difficult for injuries and diseases [24]. Shape differences between sexes may play a role in the prevalence of osteoarthritis [4,25,26,27,28] and influence the wrist biomechanics, such as kinematics [4,29] and grip strength [4,30,31]. Finally, this knowledge is required to guide the design of well-functioning biomedical devices for improved patient care. This study aimed to comprehensively and automatically characterize the wrist morphology under sex-specific aspects and to provide carpal bone correlations to predict anatomical parameters. We investigated the morphology of the eight carpal bones and two forearm bones under sex-specific considerations for 117 datasets taken from the OSCD [12].

The advantage of the computerized automatic analysis methods we developed is that they provide a means of describing the geometric bone parameters that is faster and much less tedious than manual outlining under standardized conditions. The advantage of this approach was that the database information was based on 3D imaging techniques, as opposed to data traditionally generated by analyzing planar X-ray images. The 3D analysis convincingly demonstrated that the bounding box borders were a reasonable characterization of the anatomy. In comparison, the accuracy of the data generated via plane radiographs depends on the orientation of the wrist as the X-ray is taken [3]. Plain radiographs are used often for shape classification or size determination of the carpal bones [3]. However, Watson et al. mentioned that the lunate morphology can be assigned incorrectly with relatively minor changes in the X-ray technique [32].

Our results showed that the carpal bones in the males were larger than those in the females, as generally reported in previous studies [3,12,33,34,35,36]. According to the boxplots, these differences were clear to likely clear in all dimensions except for Scaphoid z. Remarkably, Kivell et al. [4] reported that most of the sex-specific differences in the shape ratios can be vanished using sex-specific scaling relationships, but not the length of the scaphoid body, which corresponds approximately to our Scaphoid z. This sexual dimorphism requires additional investigations, especially concerning the clinical consequences, such as sex-specific designs or personalized therapy approaches. To emphasize the importance of this finding, the scaphoid is the leading bone in the proximal row. It is located at the most radial side of the proximal carpal row and is in contact with four other carpal bones. It bridges the proximal and distal carpal rows. It is the most frequently fractured carpal bone and presents clinical challenges that include inadequate diagnosis as well as healing [37].

The order from large to small of each carpal row was the same in males and females, indicating no dimorphism. This is supported by the bone ratios, which were similar for both sexes. This supports the findings by Crisco et al. [3] and Kivell et al. [4], who reported that sex differences primarily are caused by simple scaling. Furthermore, the width of the proximal row and the distal row was similar within each sex.

Our data suggest a wide range of ulnar bone sizes, indicated by the high standard deviations, the number of outliers, and the large sizes of the boxes and whiskers in the boxplots. This points to personalized rather than generic solutions, for example, in biomedical devices. The ulnar variance affects the amount of force transmitted to the distal radius and the triangular fibrocartilage complex (TFCC). Positive variance can lead to ulnar-sided wrist pain due to perforation of the TFCC and ulnar impaction syndrome. Negative ulnar variance can lead to increased shear forces and stress on the lunate, predisposing the lunate to injury. Increased pressure inside the bone along with increased stresses on the lunate can affect the blood supply, leading to avascular necrosis of the lunate (Kienböck’s disease = described as osteonecrosis of the lunate.).

Regression analysis was performed to determine the correlations between each carpal bone and its corresponding row. Very high and high correlations were observed in the proximal row, which could be used for the general design guidelines of modular sizes, surgical therapy planning, and reconstruction. In a pathological setting, bony measurements may be inappropriate. In such cases, the correlation information of healthy patients, as provided in this study, can be used for appropriate sizing. For example, progressed Kienböck’s disease can cause the lunate to lose its structural support and collapse. Even though the correlation coefficients of the lunate were rather low, they can be used to restore the overall dimensions of the proximal row based on information about the neighboring bones. The low correlations could be related to the different bone types of lunates: for example, Viegas et al. described two different types: type I and type II [38,39]. The difference is the number of facets and articulations. The type I lunate interacts via a single distal facet with the capitate, and the type II lunate interacts via two distal facets with an additional medial facet, which articulates with the hamate [2,38,39]. The suggestion was a relationship between type II lunate and hamate proximal pole arthritis [2]. A different kinematic of type I lunate exists compared to type II lunate during RUD [40,41]. However, in order to investigate this relationship in more detail, it would be necessary to also consider kinematic data.

The results of this study must be viewed with some limitations. The bounding box geometry is a simplification of the actual bone shapes. In general, the carpal shape is complex, and simple scaling may not accurately capture the local shape variations, which are important in the context of appropriate biomedical device designs. A more rigorous definition of the bone shape and geometry should lead to a more exact description of each bone and a more detailed understanding of how different factors change with size [3], although the approach is useful for explaining the dimensions and the size relationships of the carpal bones. In terms of accuracy, it is important to note that the carpal bones present at least four challenges for automated measurements: their size is small, their shapes are irregular and cannot be easily predicted, their composition is heterogeneous, and they are located close to each other [24]. Finally, ethnic differences could not be analyzed due to the lack of information in the database.

## 5. Conclusions

This study comprehensively characterized the different carpal bone sizes under sex-specific aspects for a larger number of cases and provided regression plots and equations to predict individual carpal bone sizes from the proximal and distal row dimensions as a guide for anatomically guided orthopedic biomedical device designs and therapy planning. We showed that sex-specific differences exist and that there is a need to consider individual parameters, particularly of the scaphoid and ulna. Our findings on the morphological correlations may give new insights into (healthy) bony constellations to surgeons and manufacturers. Furthermore, recognizing the dimorphism in the carpal sizes could help to better tailor patient treatment of the wrist in the future.

## Figures and Tables

**Table 1:** The following table shows the carpal bone sizes [mm] including mean and SD for the x, y, and z bounding box dimensions of our study and Crisco et al. [3] (reprinted with permission from Elsevier). NB: We defined the axes along the anatomical directions whereas Crisco et al. used the principal axes. The information was separated between males and females and also summarized for both sexes (M + F).

		Scaphoid			Lunate			Triquetrum			Pisiform			Trapezium			Trapezoid			Capitate			Hamate		
		X	Y	Z	X	Y	Z	X	Y	Z	X	Y	Z	X	Y	Z	X	Y	Z	X	Y	Z	X	Y	Z
Male n = 55	Mean	23.30	18.23	23.07	15.27	19.13	19.43	17.97	15.27	15.37	13.50	11.56	12.21	17.23	17.63	23.11	15.36	14.12	18.91	24.02	17.76	24.33	25.56	17.85	23.70
	SD	2.74	1.67	3.34	1.72	1.98	1.41	1.91	1.37	1.54	1.17	1.15	1.30	2.38	1.68	1.78	1.69	1.44	2.22	2.28	1.68	3.27	2.32	1.43	2.32
Male [3] n = 14	Mean	29.30	17.80	14.10	20.90	20.10	14.40	20.90	14.90	12.60	15.70	12.30	10.00	25.40	17.50	16.10	20.60	15.50	12.30	28.00	20.80	16.00	27.50	23.00	16.90
	SD	2.70	1.20	0.90	2.20	1.80	1.30	1.80	0.70	0.90	1.40	1.30	1.20	1.80	1.80	1.80	1.40	0.80	0.70	1.80	1.70	1.60	1.90	1.80	1.20
Female n = 62	Mean	19.99	15.33	20.69	13.12	16.21	16.64	15.97	13.89	13.75	11.81	10.29	10.90	15.07	15.73	20.27	13.56	12.42	16.91	21.39	15.69	20.89	22.99	15.81	21.08
	SD	2.22	1.34	3.37	1.30	1.19	1.05	1.58	1.44	1.35	1.24	0.95	1.32	1.99	1.20	1.71	1.59	1.32	2.12	2.31	1.18	2.55	1.89	0.90	2.30
Female [3] n = 14	Mean	24.80	15.30	12.20	18.00	16.90	11.90	18.50	13.30	10.80	13.70	10.70	8.90	21.80	15.80	13.10	18.00	13.30	11.10	24.60	18.20	13.90	24.70	20.10	15.00
	SD	1.60	1.50	0.60	1.10	0.80	0.80	1.30	0.60	0.70	1.40	1.00	0.70	1.80	1.50	1.20	0.90	1.20	0.80	1.10	1.00	0.80	1.40	0.80	0.90
M + F n = 117	Mean	21.55	16.70	21.81	14.13	17.58	17.95	16.91	14.54	14.52	12.61	10.89	11.52	16.08	16.63	21.60	14.41	13.22	17.85	22.62	16.66	22.51	24.20	16.77	22.31
	SD	2.98	2.09	3.56	1.85	2.17	1.86	2.01	1.57	1.66	1.47	1.23	1.46	2.44	1.73	2.25	1.87	1.62	2.39	2.64	1.77	3.37	2.46	1.56	2.65
M + F [3] n = 28	Mean	27.00	16.50	13.10	19.40	18.50	13.20	19.70	14.10	11.70	14.70	11.50	9.50	23.60	16.60	14.60	19.30	14.40	11.70	26.30	19.50	15.00	26.10	21.60	16.00
	SD	3.10	1.80	1.20	2.30	2.20	1.70	2.00	1.00	1.20	1.70	1.40	1.10	2.50	1.80	2.20	1.80	1.50	1.00	2.30	1.90	1.60	2.20	2.00	1.40

**Table 2:** The following table shows the width (y) and height (z) of the bones of the forearm, and the summarized length (x), width (y), and height (z) of the proximal and distal carpal bone row in [mm]. The information was also separated between males and females.

	Radius y	Radius z	Ulna y	Ulna z	Proximal Row x	Proximal Row y	Proximal Row z	Distal Row x	Distal Row y	Distal Row z
Male										
Mean	35.08	26.47	21.64	18.60	25.75	44.64	25.01	29.61	43.22	28.28
SD	2.30	2.25	4.26	3.92	2.40	3.08	2.72	2.29	2.79	2.39
Female										
Mean	30.19	22.84	18.87	16.24	23.19	39.09	22.20	27.01	38.18	24.83
SD	1.91	2.27	4.51	4.27	2.14	2.43	2.81	2.04	2.22	1.85

**Table 3:** The following table shows the ratio for the carpal bones in the x-direction to the y- and z-directions of the same bone.

	Scaphoid x/Scaphoid y		Scaphoid x/Scaphoid z		Lunate x/Lunate y		Lunate x/Lunate z		Triquetrum x/Triquetrum y		Triquetrum x/Triquetrum z		Pisiform x/Pisiform y		Pisiform x/Pisiform z		Trapezium x/Trapezium y		Trapezium x/Trapezium z		Trapezoid x/Trapezoid y		Trapezoid x/Trapezoid z		Capitate x/Capitate y		Capitate x/Capitate z		Hamate x/Hamate y		Hamate x/Hamate z	
	Female	Male	Female	Male	Female	Male	Female	Male	Female	Male	Female	Male	Female	Male	Female	Male	Female	Male	Female	Male	Female	Male	Female	Male	Female	Male	Female	Male	Female	Male	Female	Male
Mean	1.31	1.29	1.00	1.04	0.81	0.80	0.79	0.79	1.16	1.19	1.17	1.18	1.15	1.17	1.09	1.12	0.96	0.98	0.75	0.75	1.10	1.10	0.82	0.83	1.37	1.36	1.04	1.01	1.46	1.44	1.10	1.09
SD	0.16	0.17	0.24	0.24	0.11	0.09	0.07	0.08	0.14	0.15	0.15	0.16	0.11	0.11	0.13	0.14	0.10	0.11	0.09	0.11	0.16	0.15	0.18	0.17	0.15	0.14	0.20	0.20	0.11	0.15	0.15	0.16

## Data Availability

Data are contained within the article.
